# The efficacy and safety of paclitaxel and carboplatin with versus without bevacizumab in patients with non-small-cell lung cancer: a systematic review and meta-analysis

**DOI:** 10.18632/oncotarget.23657

**Published:** 2017-12-23

**Authors:** Shiqian Han, Yuanduo Hong, Tingting Liu, Na Wu, Zhijia Ye

**Affiliations:** ^1^ Institute of Tropical Medicine, Third Military Medical University, Chongqing 400038, China; ^2^ Battalion 17 of Students, College of Preventive Medicine, Third Military Medical University, Chongqing 400038, China; ^3^ Department of Epidemiology, Third Military Medical University, Chongqing 400038, China

**Keywords:** bevacizumab, paclitaxel, carboplatin, non-small cell lung cancer, safety

## Abstract

**Objectives:**

To investigate the efficacy and safety of Bevacizumab (Bev) used in combination with paclitaxel and carboplatin (PC), compared with PC alone in patients with advanced non-small-cell lung cancer (NSCLC).

**Materials and Methods:**

We searched the PubMed, EMBASE, Cochrane Central Register of Controlled Trials and Chinese Biomedical Literature electronic databases, to identify randomized controlled trials of PC plus Bev versus PC alone for the treatment of NSCLC. The meta-analysis was performed using Reviewer Manager Version 5.3 software provided by the Cochrane Collaboration. The primary endpoint was progression-free survival (PFS), and the secondary endpoints were overall survival (OS), objective response rate (ORR), the incidence of severe adverse events and treatment-related deaths.

**Results:**

The final analysis included 5 trials with a total of 1486 patients. Compared with PC alone, the regimen of PC plus Bev resulted in significantly longer PFS (HR = 0.57; 95% CI = 0.46 to 0.71; *p* < 0.00001), longer OS (HR = 0.81; 95% CI = 0.71 to 0.92; *p* = 0.0009) and higher response rates (RR = 2.06; 95% CI = 1.73 to 2.44; *p* < 0.00001). However, grade ≥ 3 neutropenia, haemoptysis, hypertension, proteinuria and bleeding events were more common among patients who received Bev, and these patients also experienced increased rates of treatment-related death.

**Conclusions:**

Compared with PC alone, the combination of PC with Bev could prolong PFS, OS and RR for patients with advanced non-squamous NSCLC. However, this combination could lead to a higher toxicity profile. Therefore, the benefits and risks should be considered before making treatment decisions.

## INTRODUCTION

Non-small-cell lung cancer (NSCLC) is the most common type of lung cancer and the leading cause of cancer-related death in the world [1]. Patients with advanced or metastatic NSCLC have a poor prognosis. Chemotherapy with doublet platinum-based compounds is recommended as the first-line treatment for advanced NSCLC patients, but the treatment benefit is limited [2, 3]. The Eastern Cooperative Oncology Group (ECOG) conducted a randomized study to compare the efficacy and safety of four common platinum-based treatments (cisplatin and gemcitabine, cisplatin and docetaxel, paclitaxel and carboplatin [PC] or paclitaxel and cisplatin). No significant difference in overall survival (OS) was found. Although these regimens demonstrate modest progress in terms of outcome, with a median survival time of approximately 8 months [4], more effective and/or better tolerated agents for advanced NSCLC are needed.

Given data show that [5] expression of vascular endothelial growth factor (VEGF) increases during the development of various tumor types and plays a critical role in tumor angiogenesis after binding with VEGF receptor (VEGFR). Bevacizumab (Bev) is a recombinant humanized monoclonal antibody that can recognize VEGFR and block the biological activity of VEGF. Bev was approved for use in patients with advanced NSCLC by the U.S. Food and Drug Administration (FDA) [6]. In Europe, the regimen of Bev plus a platinum-based two-drug regimen was also approved for first-line treatment in patients with advanced NSCLC [7].

Increasing numbers of clinical trials have been conducted with Bev for the treatment of patients with advanced NSCLC since 2004. However, the benefits and safety of Bev remain controversial [8, 9]. All the published meta-analyses [10–12] have compared the efficacy and toxicity of Bev used in combination with platinum-based chemotherapy, with those of chemotherapy alone in patients with advanced NSCLC. The different combinations of platinum-based chemotherapy, may lead to significant heterogeneity and different results (complete remission, partial remission, or stable disease) among the studies. This heterogeneity stresses the importance of further assessing specific chemotherapy regimens. Therefore, the aim of our meta-analysis is to evaluate the efficacy and safety of PC with or without Bev in patients with advanced NSCLC by collecting data from randomized control trials and phase II or III trials. We have chosen the PC regimen because it is effective and less toxic than other treatment regimens [4].

## MATERIALS AND METHODS

### Search strategy

The meta-analysis was performed according to the Preferred Reporting Items for Systematic Reviews and Meta-Analyses (PRISMA) guidelines [13]. A wide search of the primary electronic databases of interest was conducted, including PubMed, EMBASE, Cochrane Central Register of Controlled Trials and the Chinese Biomedical Literature (CBM) databases. Furthermore, the abstracts and virtual meeting presentations published in the proceedings of the American Society of Clinical Oncology (ASCO), American Society of Hematology (ASH), the European Society for Medical Oncology (ESMO) and International Association for the Study of Lung Cancer (IASLC) were also searched. Both published and unpublished studies were included to minimize publication bias [14]. The search terms used to identify the related studies were “bevacizumab,” “carboplatin,” “paclitaxel,” “lung,” “neoplasms,” “randomized controlled trial,” and “RCT.”

All references were scanned and eligible trials were selected independently by two of the researchers (S.H. and Y.H.). The search included literature published or presented up to May 2017.

### Inclusion and exclusion criteria

The eligible studies included randomized, parallel design, or clinical trials comparing PC with or without Bev (dose: 15 mg/kg) as a first-line therapy for patients with untreated locally advanced, recurrent or previously metastatic NSCLC. Studies that were not randomized clinical trials (RCTs) or were reviews of this topic were excluded from this meta-analysis.

### Data extraction

Using standard data extraction forms, two reviewers (S.H. and Y.H.) independently extracted data from all the included studies. We extracted publication information (first author's name, publication year, country, study design), participant characteristics (mean age of participants, gender, sample size, intervention and comparisons, histology, primary endpoint), and endpoints (progression-free survival(PFS), OS objective response rate [ORR], incidence of Common Toxicity Criteria scale grade 3/4 toxicities and treatment related mortality). Any differences in opinions were resolved by a third reviewer (N.W.).

The primary endpoint of this study was PFS, which was the time between random assignment and the first report of disease progression, all-cause mortality, or date of the last follow-up visit for patients who were alive without progression. The secondary endpoint was OS, which was defined as the time between random assignment and either death from any cause or the date of the final follow-up in the case of survival. The ORR was defined as the sum of the partial and complete response rates [15]. Addtionally, the adverse drug reaction rate was graded according to the Common Toxicity Criteria version 3. We also considered the treatment related mortality for the analyses.

The hazard ratios (HRs) and their 95% confidence interval (CI) of the time-to-event data (PFS and OS) were directly extracted from the original studies or they were estimated indirectly using either the reported number of events and the corresponding *p*-value for the log-rank statistics, or by using the Engauge Digitizer V4.1 screenshot tool (M. Mitchell, Engauge Digitizer, http://digitizer.sourceforge.net) and reading off Kaplan-Meier curves as described by Parmar et al [16]. The calculations were performed using the spreadsheet proposed by Tierney et al. [17]. For this analysis, we enlarged the original survival curves from the previous trial, and extracted the exact values by digitizing the data points in an image file after manually setting the axis coordinates.

### Quality assessment

To assess the quality of the included trials, two investigators (S.H. and Y.H.) independently used the Cochrane Collaboration tool [18], and the following items were extracted: random sequence generation, allocation concealment, blinding of participants and personnel, blinding of outcomes assessment, incomplete PFS, OS and ORR data, selective reporting and other biases. When there was insufficient information to evaluate the quality of the trials, it was defined as unclear (i.e., uncertain risk of bias).

### Statistical analysis

The time-to-event outcomes were compared using an HR, and dichotomous data were compared using the risk ratio (RR) [16]. The respective 95%CIs were calculated for each estimate and presented in forest plots. The pooled HR or RR, which is represented by a solid diamond at the bottom of the forest plot (the width of which represents the 95% CI) is the best estimate of the true (pooled) outcome. The effect of the treatment for each study is expressed as the ratio of the Bev plus PC arm over the PC alone arm.

Statistical heterogeneity among trials was tested using the Chi-square test [19], and was expressed by the I2 index, which describes the proportion of total variation across the studies that results from heterogeneity rather than chance [20]. When significant heterogeneity was detected (I2 > 50%), a possible explanation was considered and a random-effects model was applied. Otherwise, the fixed-effects model was used to pool the data. All meta-analyses were performed using RevMan 5.3 software (RevMan software, version 5.3, Cochrane Collaboration, Oxford, UK). Egger's test and Begg's test were used to assess the possibility of publication and selection bias [21, 22]. A forest plot was used to display the results.

## RESULTS

### Description of studies

**Figure 1:**
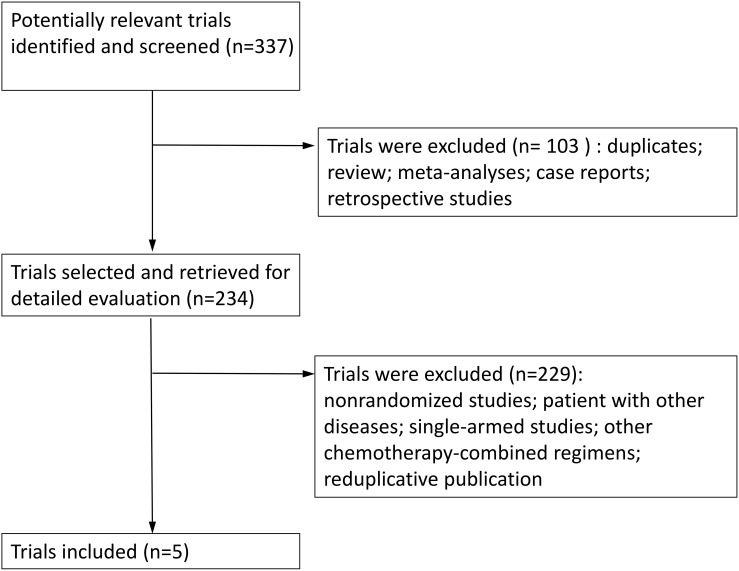
Flow of identification and inclusion of trials

**Figure 2:**
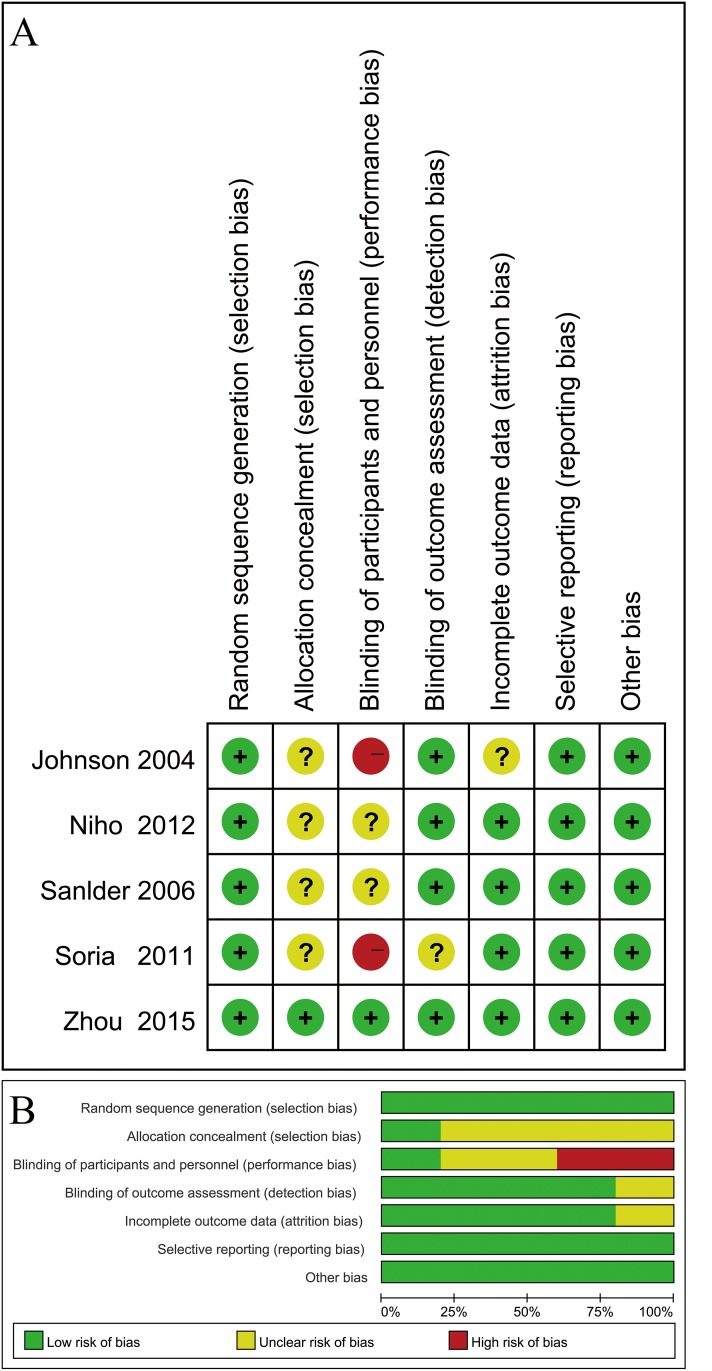
Evaluation of study quality (**A**) Risk of bias for each included RCT. (**B**) Bar chart comparing percentage risk of bias for each included RCT.

A total of 337 trials were retrieved after the initial literature search, with 72 duplicates. After reading the title and abstract of the studies, 103 trials were excluded; 234 potentially relevant full-text trials were reviews, and we found five RCTs (1486 patients) that compared PC with or without Bev (dose: 15 mg/kg) for locally advanced (stage IIIB), recurrent or metastatic (stage IV) NSCLC [8, 23–26]. We created a diagram to represent the flow of the selection and inclusion of trials (Figure 1). The characteristics of the included trials and the evaluation of study quality are shown in Table 1 and Figure 2.

**Table 1:** Characteristics of RCTs included in the meta-analysis

study	year	region	trial phase	participants	intervention and comparisons	patients enrolled	Histology	primary endpoint
Johnson	2004	USA	II	99	C:CP T:CP+BEV(7.5 mg/kg) T:CP+BEV(15 mg/kg)	32 32 35	adenocarcinoma, large cell carcinoma, squamous cell carcinoma, other	time to disease progression and tumor response rate
Sandler	2006	USA	III	878	C:CP T:CP+BEV(15 mg/kg)	444 434	adenocarcinoma, large cell carcinoma, bronchoalveolar carcinoma, other	overall survival
Soria	2011	Europe	II	85	C:CP T:CP+BEV(15 mg/kg)	41 44	adenocarcinoma, bronchoalveolar carcinoma, large cell carcinoma, other	objective response rate
Niho	2012	Japan	II	180	C:CP T:CP+BEV(15 mg/kg)	59 121	adenocarcinoma, large cell carcinoma, other	progression-free survival
Zhou	2015	China	III	276	C:CP T:CP+BEV(15 mg/kg)	138 138	adenocarcinoma, large cell carcinoma, mixed cell carcinoma	progression-free survival

The inclusion of five RCTs included three phase II and two phase III clinical trials. Johnson et al's phase II trial included patients with squamous cell histology, which showed a greater tendency to cavitate than adenocarcinoma, and may have led to a greater incidence of fatal bleeding. Therefore, squamous cell histology became an exclusion criterion in the other trials of our meta-analysis. Patients received paclitaxel (200 mg/m^2^) and carboplatin (an area under the concentration–time curve of 6.0mg/mL· min) or PC plus Bev at a dose of 15 mg/kg given intravenously on day 1 of each cycle. Chemotherapy was repeated every 21 days for 6 cycles unless there was evidence of disease progression, unacceptable toxicity, or death. In Zhou et al›s phase III trial, patients were administered paclitaxel at a dose of 175 mg/m^2^, which reflected the approved dose in China.

According to Begg's test and Egger's test, there was no significant publication bias with respect to any of the end points.

### Progression-free survival

**Figure 3:**
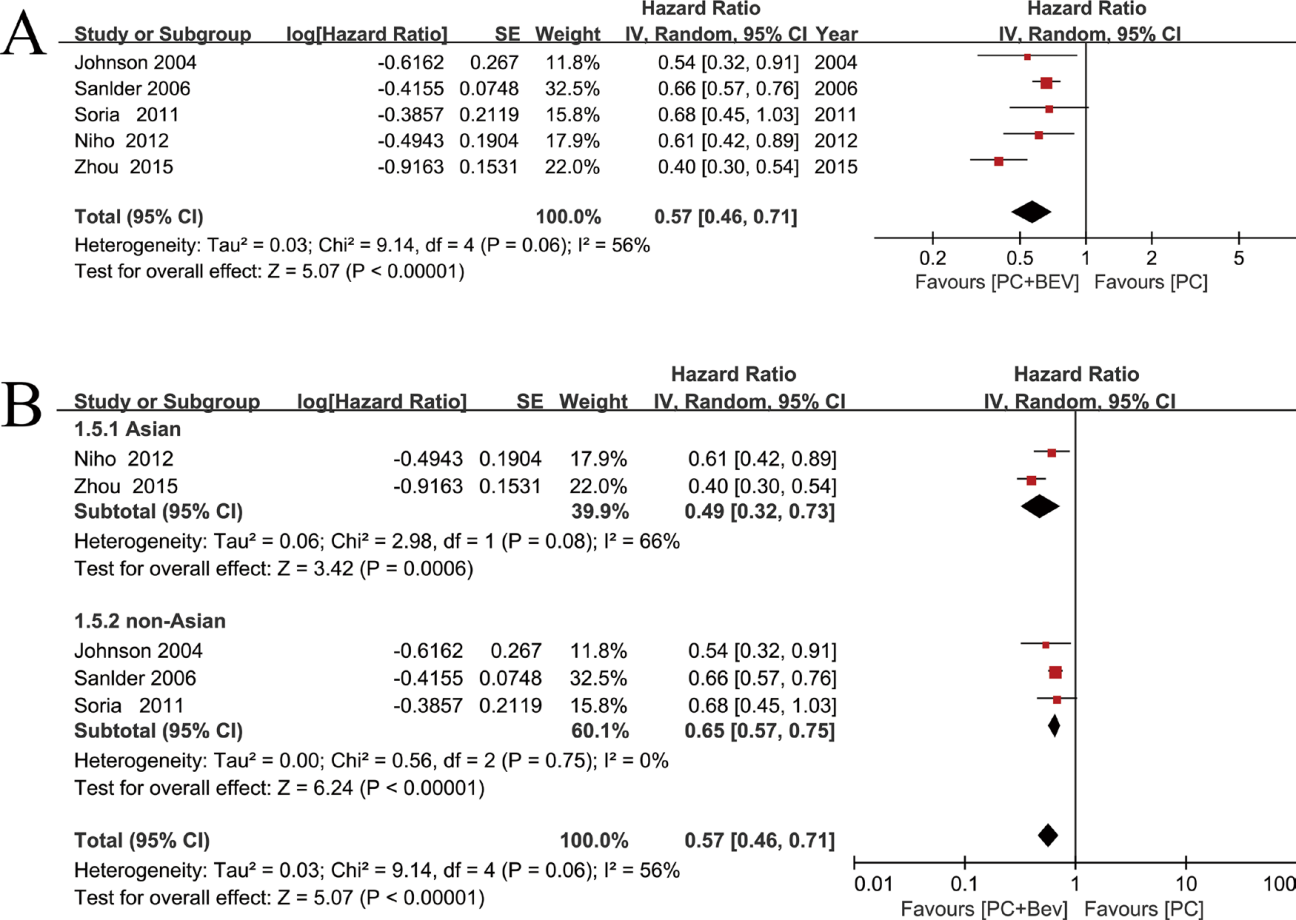
Meta-analysis of progression-free survival (**A**) Forest plot of PFS. (**B**) Forest plot of PFS in the subgroup analysis: Asian, non-Asian.

PFS was prolonged in patients treated who were with PC plus Bev, compared with PC, with an estimated HR of 0.57 (random effects: 95% CI = 0.46–0.71, *p* < 0.01; I^2^ = 56%, *p* = 0.06) (Figure 3A). High heterogeneity was observed in the Asian subgroup (I^2^ = 66%), whereas there was no heterogeneity in the non-Asian subgroup (I^2^ = 0) (Figure 3B). We tried to compared the two trials in the Asian subgroup, and found that Zhou et al's phase III trial was well balanced regarding the patient characteristics. There was a higher proportion of female patients and patients who had never smoked than in the population of the other trial.

### Overall survival

**Figure 4:**
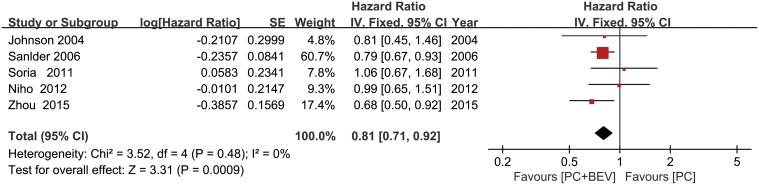
Meta-analysis of overall survival

The five included trials all reported OS. The HR for the OS favored Bev combined with PC (fixed effect: HR = 0.81; 95% CI = 0.71–0.92; *p* < 0.01), without significant heterogeneity (I^2^ = 0%; *p* = 0.48) (Figure 4) among the trials, and HR was calculated using a fixed effects model. There was also no significant heterogeneity (I^2^ = 15%, *P* = 0.32)with regarding the effect of Bev on the OS after excluding the study published by Johnson et al., which was the only study that included patients with squamous cell histology.

### Overall response rates

**Figure 5:**
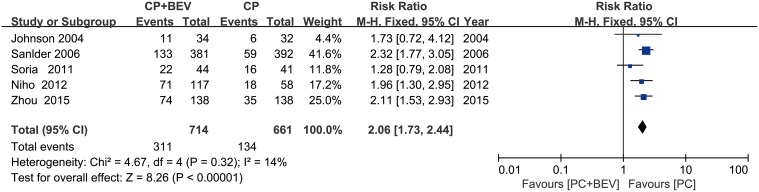
Meta-analysis of overall response rates

The fixed-effects model evaluation (χ^2^ = 4.67; *p* = 0.32, I^2^ = 14%), including 1,486 patients, showed an increased response rate in the Bev plus PC versus the PC along group (RR = 2.06, 95% CI = 1.73–2.44) (Figure 5).

### Toxicities and safety

**Figure 6:**
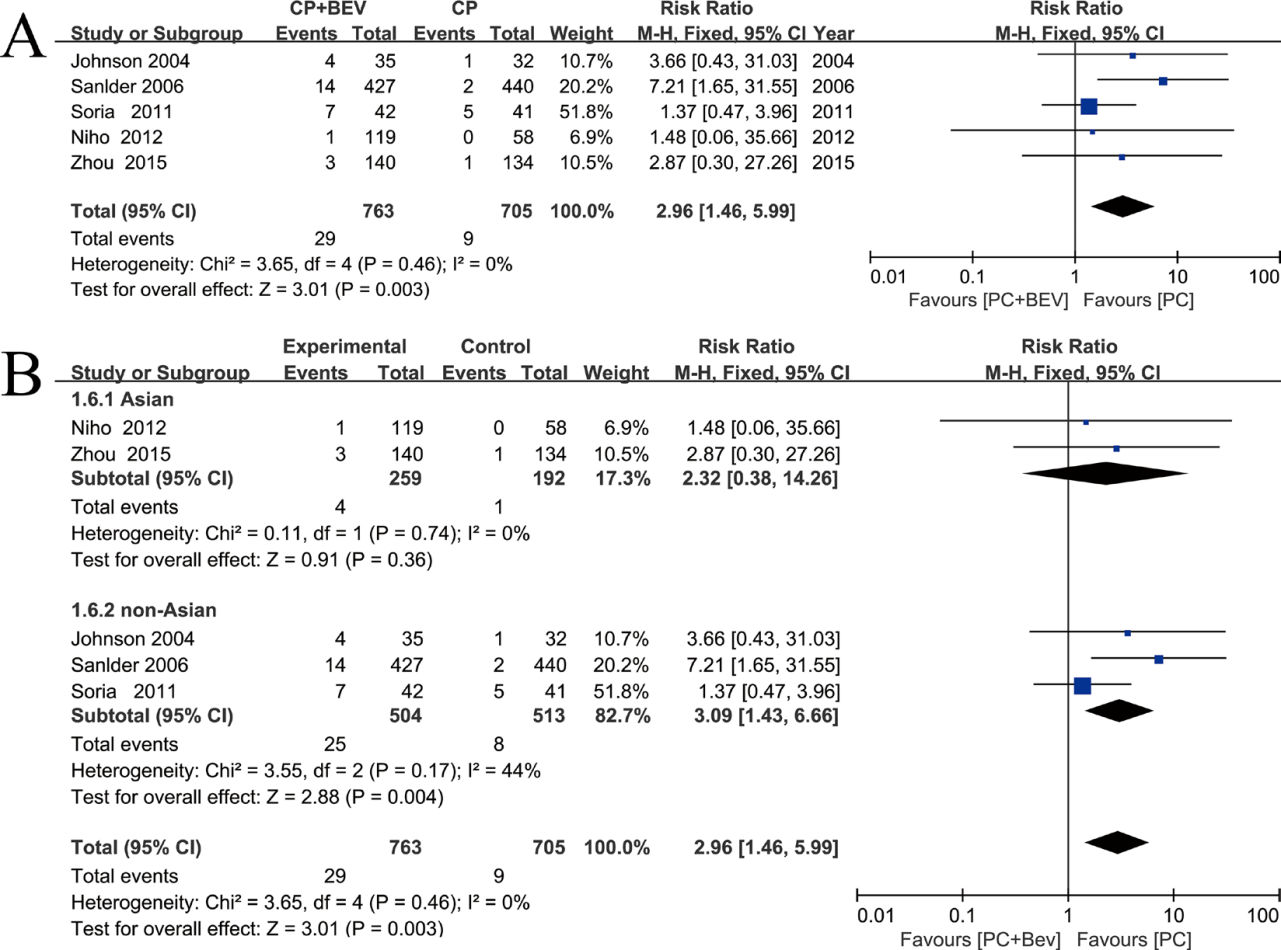
Meta-analysis of treatment-related deaths (**A**) Forest plot of treatment-related deaths. (**B**) Forest plot of treatment-related deaths in the subgroup analysis: Asian, non-Asian.

Bev showed a significant increase in treatment-related deaths in patients with NLCLC (fixed effect: RR = 2.96; 95% CI = 1.46–5.99; *p* = 0.003) (Figure 6A). We performed subgroup analyses of Asian groups and non-Asian groups. Treatment-related deaths were significantly increased in the non-Asian groups (fixed effect: RR = 3.09; 95% CI = 1.43–6.66; *p* = 0.004). The Asian subgroup analyses did not yield similar results (fixed effect: RR = 2.32; 95% = 0.38–14.26; *p* = 0.74) (Figure 6B).

**Figure 7:**
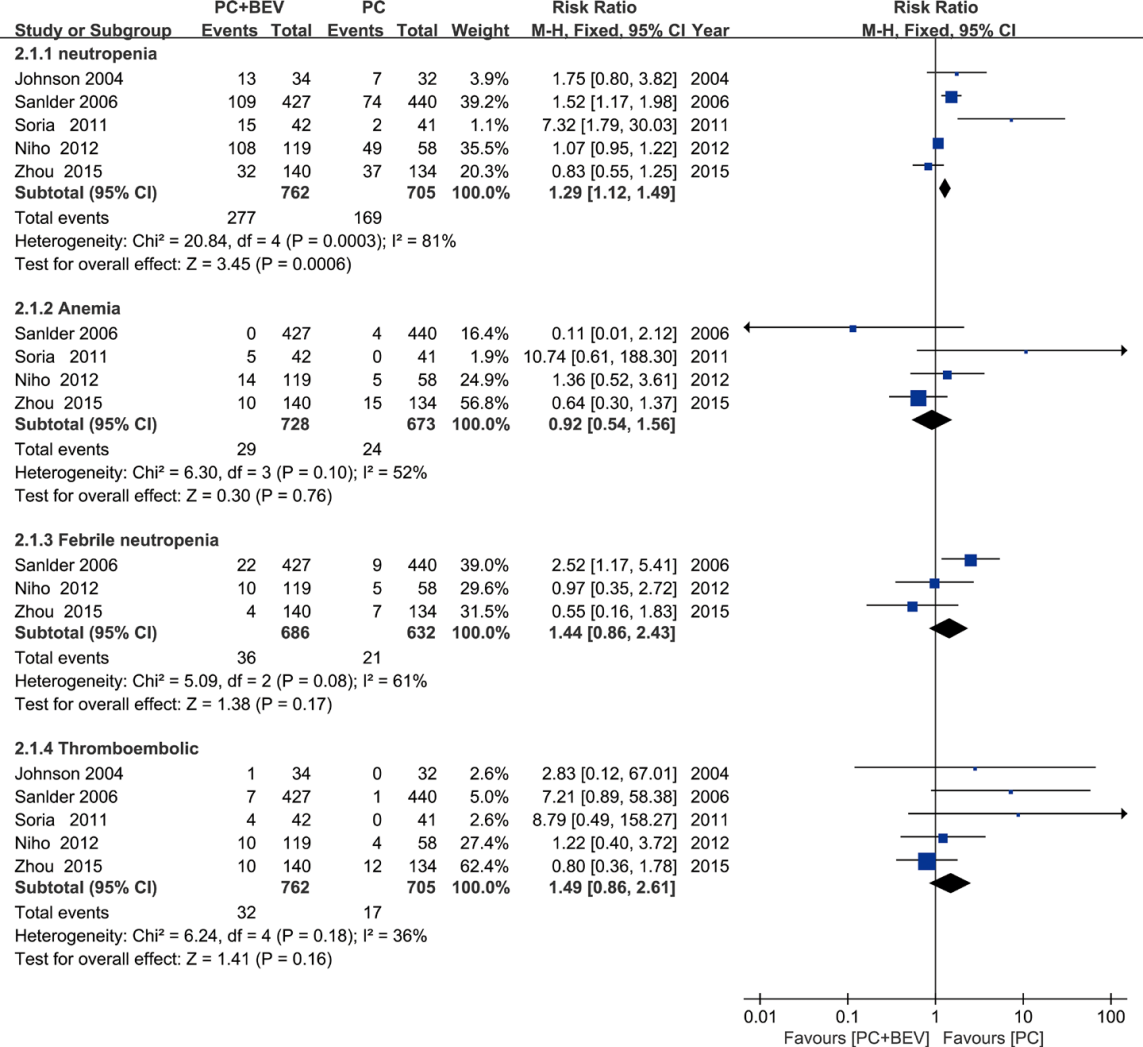
Meta-analysis of haematologic toxicities

According to the haematological toxicities (grade 3/4), the group that received PC plus Bev had higher rates of neutropenia (fixed effect: RR = 1.29; 95% CI = 1.12–1.49; *p* = 0.0006). The proportions of febrile anemia (fixed effect: RR = 0.92; 95% CI = 0.54–1.56; *p* = 0.76), febrile neutropenia (fixed effect: RR = 1.44; 95% CI = 0.86–2.43; *p* = 0.17) and thrombocytopenia (fixed effect: RR = 1.49; 95% CI = 0.86–2.61; *p* = 0.16) were similar (Figure 7).

**Figure 8:**
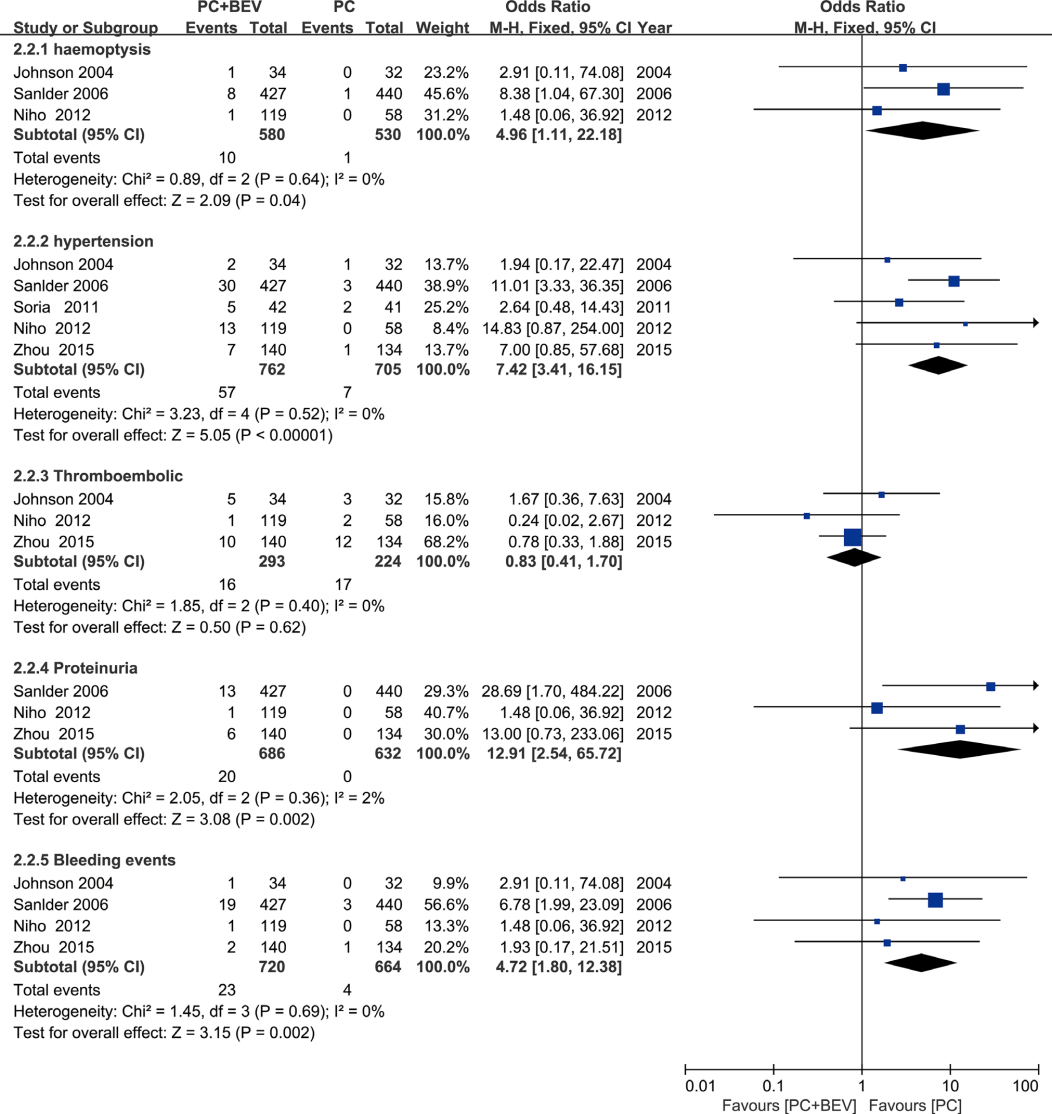
Meta-analysis of non-haematologic toxicities

The non-haematologic toxicities were also more frequent for patients receiving PC plus Bev. These toxicities included haemoptysis(fixed effect: RR = 4.87; 95%CI = 1.13–20.90; *p* = 0.03), hypertension (fixed effect: RR = 6.89; 95% CI = 3.21–14.79; *p* < 0.00001), proteinuria (fixed effect: RR = 12.58; 95% CI = 2.61–60.57; *p* = 0.002) and bleeding events (fixed effect: RR = 4.59; 95% CI = 1.78–11.80; *p* = 0.002). There was no difference in the proportion of patients with thrombocytopenia (fixed effect: RR = 0.85; 95% CI = 0.44–1.62; *p* = 0.61) (Figure 8).

## DISCUSSION

As the first Food and Drug Administration (FDA) approved anti-vascular VEGF drug, Bev can inhibit angiogenesis, reduce vascular permeability and block delivery of nutrients via the blood vessels that a tumor needs to develop [27]. Several studies have explored safe doses of Bev. Gordon et al's phase I trial [28] examined a Bev dose range from 0.1–10 mg/kg, and found that VEGF was not detected in the blood when the dose was 0.3 mg/kg. Cobleign et al's phase II trial [29] evaluated the range of 3–20 mg/kg. Most patients achieved a partial response after taking the 10mg/kg dose of Bev, while Bev caused a variety of adverse reactions at a dose of 20 mg/kg. Johnson et al's phase III trials [23] used dose of 7.5 mg/kg and 15 mg/kg based on pharmacokinetic models, which helped determined the clinical trial dose. *In vitro* data [30, 31] showed that the low dose of Bev induced the normalization of blood vessels without affecting tumor cell viability, while high doses led to a significant reduction in the tumor growth and prolonged patient survival. Therefore, our meta-analysis examined Bev at a dose of 15 mg/kg.

Platinum-based chemotherapy regimens remain the standard of treatment for patients with advanced, unresectable or metastatic NSCLC [32]. As the second generation of non-specific anti-tumor platinum chemotherapy drugs, carboplatin interferes with DNA synthesis and causes cytotoxic effects. Both cisplatin and carboplatin are supported for the first-line treatment of advanced NSCLC [33]. Compared with cisplatin, carboplatin significantly reduces nephrotoxicity, ototoxicity, neurotoxicity and gastrointestinal adverse reactions. Paclitaxel is in the taxane family of anti-microtubule drugs and inhibits the normal function of tubulin during cell mitosis; it was approved by the FDA for the treatment of pancreatic cancer, breast cancer, and NSCLC. Thus, the PC regime is a basic program for future clinical trials of multi-drug chemotherapy.

Two well-known phase III trials, ECOG 4599 and AVAiL [8, 9], have formed the basis of the regulatory approval of Bev for the treatment of advanced NSCLC in many countries. The PFS and objective response rate are similar in both trials. However, the results in OS remain controversial. ECOG 4599 [8] demonstrated that Bev combined with the PC regimen significantly improved the OS (12.3 months vs 10.3 months, *p* = 0.003), and this regimen became the first program that increased the OS to more than one year in the history of advanced NSCLC treatment. In the AVAiL study [9], Bev combined with carboplatin and gemcitabine failed to achieve this goal (13.6 months, 13.4 months vs 13.1 months, *p* > 0.05), and patients may have received more effective post-treatment compared with the E4599 trial. Additionally, ECOG4599 found that involving paclitaxel can rapidly induce circulating endothelial progenitor cells (CEPs) and tumor homing, but gemcitabine does not [34]. Paclitaxel and Bev may exhibit synergistic effects. The aim of our meta-analysis was to evaluate the efficacy of PC with versus without Bev in patients with advanced NSCLC, and we indeed found that the addition of Bev to PC significantly improved the PFS, OS and RR when compared with PC alone.

Treatment-related death and other adverse events related to Bev treatment are a great concern. A meta-analysis in JAMA [35] showed that, compared with chemotherapy alone, Bev in combination with chemotherapy, was associated with increased treatment-related mortality for cancer patients, which is consistent with our results. Because of its unique mechanism, the adverse events of Bev are different from those of general chemotherapy. Bleeding, gastrointestinal perforation and neutropenia were especially apparent in cases of death in the Bev group; thus, patients receiving Bev should be carefully monitored. Randomized studies have evaluated the benefits and toxicities of Bev for the treatment of NSCLC. It is unclear whether the discrepancies among trials are due to racial differences. Our study found that Bev has better efficacy and an acceptable safety profile in Asian populations (Figure 3B, 6B), and the same phenomenon has been reported after pooling the data from Asian patients [36, 37]. In addition, our meta-analysis showed that Bev increased the risk of grade ≥ 3 neutropenia, haemoptysis, hypertension, proteinuria and bleeding events. However, these adverse events are manageable in clinical practice and do not require permanent suspension of Bev. No new safety concerns were identified. Bev plus PC can be considered an effective first-line treatment for advanced NSCLC when the applicable patients were carefully selected and the relevant toxic reactions were managed in a timely manner.

The 5 included studies were all RCTs, and were assessed by the Cochrane Collaboration tool. Our result showed that there was a low risk of bias in most domains except for the allocation concealment and binding. Because the outcomes (such as PFS and OS) in cancer trials are objective and are not influenced by a lack of blinding, the risk of bias was considered acceptable. The quality of the trials included in our meta-analysis was reliable and provided an evidence-based medical basis for future clinical treatment.

Our meta-analysis is the first comprehensive comparative study of PC with versus without Bev in patients with advanced NSCLC. The previously published meta-analysis assessed the efficacy and toxicity of Bev used in combination with platinum-based chemotherapy, compared with chemotherapy alone. However, different combinations of platinum-based chemotherapeutic compounds, may lead to significant heterogeneity and different results among the valuable studies. Our meta-analysis also has several limitations. First, in common with other studies [10–12, 38], our study was conducted using summary data not individual patient data from each trial [39, 40]. In addition, the sample size of patients was still small. In the future, large-scale, multicenter, long-term follow-up studies are required.

## CONCLUSIONS

Our meta-analysis demonstrated that Bev significantly prolonged the PFS, OS and RR when combined with PC as first-line therapy in patients with non-squamous advanced NSCLC. This combination caused more adverse events and slightly increased the risk of treatment-related death. Thus, Bev plus PC can be considered a good option for reasonably selected target patients. Importantly, the patient's own value, complicated diseases and expected toxicity profile should be considered before making a treatment decision.

## References

[1] Siegel RL, Miller KD, Jemal A (2016). Cancer statistics, 2016. CA: a cancer journal for clinicians.

[2] Soon YY, Stockler MR, Askie LM, Boyer MJ (2009). Duration of chemotherapy for advanced non-small-cell lung cancer: a systematic review and meta-analysis of randomized trials. J Clin Oncol.

[3] Goffin J, Lacchetti C, Ellis PM, Ung YC, Evans WK (2010). First-line systemic chemotherapy in the treatment of advanced non-small cell lung cancer: a systematic review. J Thorac Oncol.

[4] Schiller JH, Harrington D, Belani CP, Langer C, Sandler A, Krook J, Zhu J, Johnson DH (2002). Comparison of four chemotherapy regimens for advanced non-small-cell lung cancer. N Engl J Med.

[5] Kerbel RS (2008). Tumor Angiogenesis. New Engl J Med.

[6] Cohen MH, Gootenberg J, Keegan P, Pazdur R (2007). FDA drug approval summary: bevacizumab (Avastin) plus Carboplatin and Paclitaxel as first-line treatment of advanced/metastatic recurrent nonsquamous non-small cell lung cancer. Oncologist.

[7] Langer C, Soria JC (2010). The role of anti-epidermal growth factor receptor and anti-vascular endothelial growth factor therapies in the treatment of non-small-cell lung cancer. Clin Lung CanceR.

[8] Sandler A, Gray R, Perry MC, Brahmer J, Schiller JH, Dowlati A, Lilenbaum R, Johnson DH (2006). Paclitaxel–carboplatin alone or with bevacizumab for non–small-cell lung cancer. New Engl J Med.

[9] Reck M, von Pawel J, Zatloukal P, Ramlau R, Gorbounova V, Hirsh V, Leighl N, Mezger J, Archer V, Moore N (2009). Phase III trial of cisplatin plus gemcitabine with either placebo or bevacizumab as first-line therapy for nonsquamous non–small-cell lung cancer: Avail. J Clin Oncol.

[10] Lima ABC, Macedo LT, Sasse AD (2011). Addition of bevacizumab to chemotherapy in advanced non-small cell lung cancer: a systematic review and meta-analysis. Plos One.

[11] Botrel TEA, Clark O, Clark L, Paladini L, Faleiros E, Pegoretti B (2011). Efficacy of bevacizumab (Bev) plus chemotherapy (CT) compared to CT alone in previously untreated locally advanced or metastatic non-small cell lung cancer (NSCLC): Systematic review and meta-analysis. Lung Cancer.

[12] Soria J, Mauguen A, Reck M, Sandler AB, Saijo N, Johnson DH, Burcoveanu D, Fukuoka M, Besse B, Pignon J (2012). Systematic review and meta-analysis of randomised, phase II/III trials adding bevacizumab to platinum-based chemotherapy as first-line treatment in patients with advanced non-small-cell lung cancer. Ann Oncol.

[13] Moher D, Liberati A, Tetzlaff J, Altman DG, Prisma G (2009). Preferred reporting items for systematic reviews and meta-analyses: the PRISMA statement. Plos Med.

[14] Begg CB, Berlin JA (1989). Publication bias and dissemination of clinical research. J Natl Cancer Inst.

[15] Therasse P, Arbuck SG, Eisenhauer EA, Wanders J, Kaplan RS, Rubinstein L, Verweij J, Van Glabbeke M, van Oosterom AT, Christian MC (2000). New guidelines to evaluate the response to treatment in solid tumors. Journal of the National Cancer Institute.

[16] Parmar MK, Torri V, Stewart L (1998). Extracting summary statistics to perform meta-analyses of the published literature for survival endpoints. Stat Med.

[17] Tierney JF, Stewart LA, Ghersi D, Burdett S, Sydes MR (2007). Practical methods for incorporating summary time-to-event data into meta-analysis. Trials.

[18] Higgins JP, Green S (2011). Cochrane handbook for systematic reviews of interventions.

[19] DerSimonian R, Laird N (1986). Meta-analysis in clinical trials. Control Clin Trials.

[20] Higgins JP, Thompson SG, Deeks JJ, Altman DG (2003). Measuring inconsistency in meta-analyses. BMJ.

[21] Begg CB, Mazumdar M (1994). Operating characteristics of a rank correlation test for publication bias. Biometrics.

[22] Egger M, Davey SG, Schneider M, Minder C (1997). Bias in meta-analysis detected by a simple, graphical test. BMJ.

[23] Johnson DH, Fehrenbacher L, Novotny WF, Herbst RS, Nemunaitis JJ, Jablons DM, Langer CJ, DeVore RR, Gaudreault J, Damico LA, Holmgren E, Kabbinavar F (2004). Randomized phase II trial comparing bevacizumab plus carboplatin and paclitaxel with carboplatin and paclitaxel alone in previously untreated locally advanced or metastatic non-small-cell lung cancer. J Clin Oncol.

[24] Soria J, Márk Z, Zatloukal P, Szima B, Albert I, Juhász E, Pujol J, Kozielski J, Baker N, Smethurst D (2011). Randomized phase II study of dulanermin in combination with paclitaxel, carboplatin, and bevacizumab in advanced non–small-cell lung cancer. J Clin Oncol.

[25] Niho S, Kunitoh H, Nokihara H, Horai T, Ichinose Y, Hida T, Yamamoto N, Kawahara M, Shinkai T, Nakagawa K (2012). Randomized phase II study of first-line carboplatin-paclitaxel with or without bevacizumab in Japanese patients with advanced non-squamous non-small-cell lung cancer. Lung Cancer.

[26] Zhou C, Wu Y, Chen G, Liu X, Zhu Y, Lu S, Feng J, He J, Han B, Wang J (2015). BEYOND: a randomized, double-blind, placebo-controlled, multicenter, phase III study of first-line carboplatin/paclitaxel plus bevacizumab or placebo in Chinese patients with advanced or recurrent nonsquamous non–small-cell lung cancer. J Clin Oncol.

[27] Ferrara N, Hillan KJ, Gerber HP, Novotny W (2004). Discovery and development of bevacizumab, an anti-VEGF antibody for treating cancer. Nat Rev Drug Discov.

[28] Gordon MS, Margolin K, Talpaz M, Sledge GW, Holmgren E, Benjamin R, Stalter S, Shak S, Adelman DC (2001). Phase I safety and pharmacokinetic study of recombinant human anti-vascular endothelial growth factor in patients with advanced cancer. J Clin Oncol.

[29] Cobleigh MA, Langmuir VK, Sledge GW, Miller KD, Haney L, Novotny WF, Reimann JD, Vassel A (2003). A phase I/II dose-escalation trial of bevacizumab in previously treated metastatic breast cancer.

[30] von Baumgarten L, Brucker D, Tirniceru A, Kienast Y, Grau S, Burgold S, Herms J, Winkler F (2011). Bevacizumab has differential and dose-dependent effects on glioma blood vessels and tumor cells. Clin Cancer Res.

[31] Pechman KR, Donohoe DL, Bedekar DP, Kurpad SN, Hoffmann RG, Schmainda KM (2011). Characterization of bevacizumab dose response relationship in U87 brain tumors using magnetic resonance imaging measures of enhancing tumor volume and relative cerebral blood volume. J Neuro-Oncol.

[32] D’Addario G, Pintilie M, Leighl NB, Feld R, Cerny T, Shepherd FA (2005). Platinum-based versus non-platinum-based chemotherapy in advanced non-small-cell lung cancer: a meta-analysis of the published literature. J Clin Oncol.

[33] Klastersky J, Awada A (2012). Milestones in the use of chemotherapy for the management of non-small cell lung cancer (NSCLC). Crit Rev Oncol Hematol.

[34] Shaked Y, Henke E, Roodhart JM, Mancuso P, Langenberg MH, Colleoni M, Daenen LG, Man S, Xu P, Emmenegger U, Tang T, Zhu Z, Witte L (2008). Rapid chemotherapy-induced acute endothelial progenitor cell mobilization: implications for antiangiogenic drugs as chemosensitizing agents. Cancer Cell.

[35] Ranpura V, Hapani S, Wu S (2011). Treatment-related mortality with bevacizumab in cancer patients: a meta-analysis. JAMA.

[36] Tsai CM, Au JS, Chang GC, Cheng AC, Zhou C, Wu YL (2011). Safety and efficacy of first-line bevacizumab with chemotherapy in Asian patients with advanced nonsquamous NSCLC: results from the phase IV MO19390 (SAiL) study. J Thorac Oncol.

[37] Mok TS, Hsia TC, Tsai CM, Tsang K, Chang GC, Chang JW, Sirisinha T, Sriuranpong V, Thongprasert S, Chua DT, Moore N, Manegold C (2011). Efficacy of bevacizumab with cisplatin and gemcitabine in Asian patients with advanced or recurrent non-squamous non-small cell lung cancer who have not received prior chemotherapy: A substudy of the Avastin in Lung trial. Asia-Pacific Journal of Clinical Oncology.

[38] Ahmadizar F, Onland-Moret NC, De Boer A, Liu G, Maitland-Van Der Zee AH (2015). Efficacy and safety assessment of the addition of bevacizumab to adjuvant therapy agents in cancer patients: a systematic review and meta-analysis of randomized controlled trials. Plos One.

[39] Pignon JP, Bourhis J (1995). Meta-analysis of chemotherapy in head and neck cancer: individual patient data vs literature data. Brit J Cancer.

[40] Riley RD, Lambert PC, Abo-Zaid G (2010). Meta-analysis of individual participant data: rationale, conduct, and reporting. Bmj.

